# Supplementary figures and images for: Wheat inositol pyrophosphate kinase TaVIH2-3B modulates cell-wall composition and drought tolerance in Arabidopsis
Source: BMC Biol. 2021 Dec 11;19:261. doi: 10.1186/s12915-021-01198-8 (PMC8665518; doi:10.1186/s12915-021-01198-8)

## Slide 1
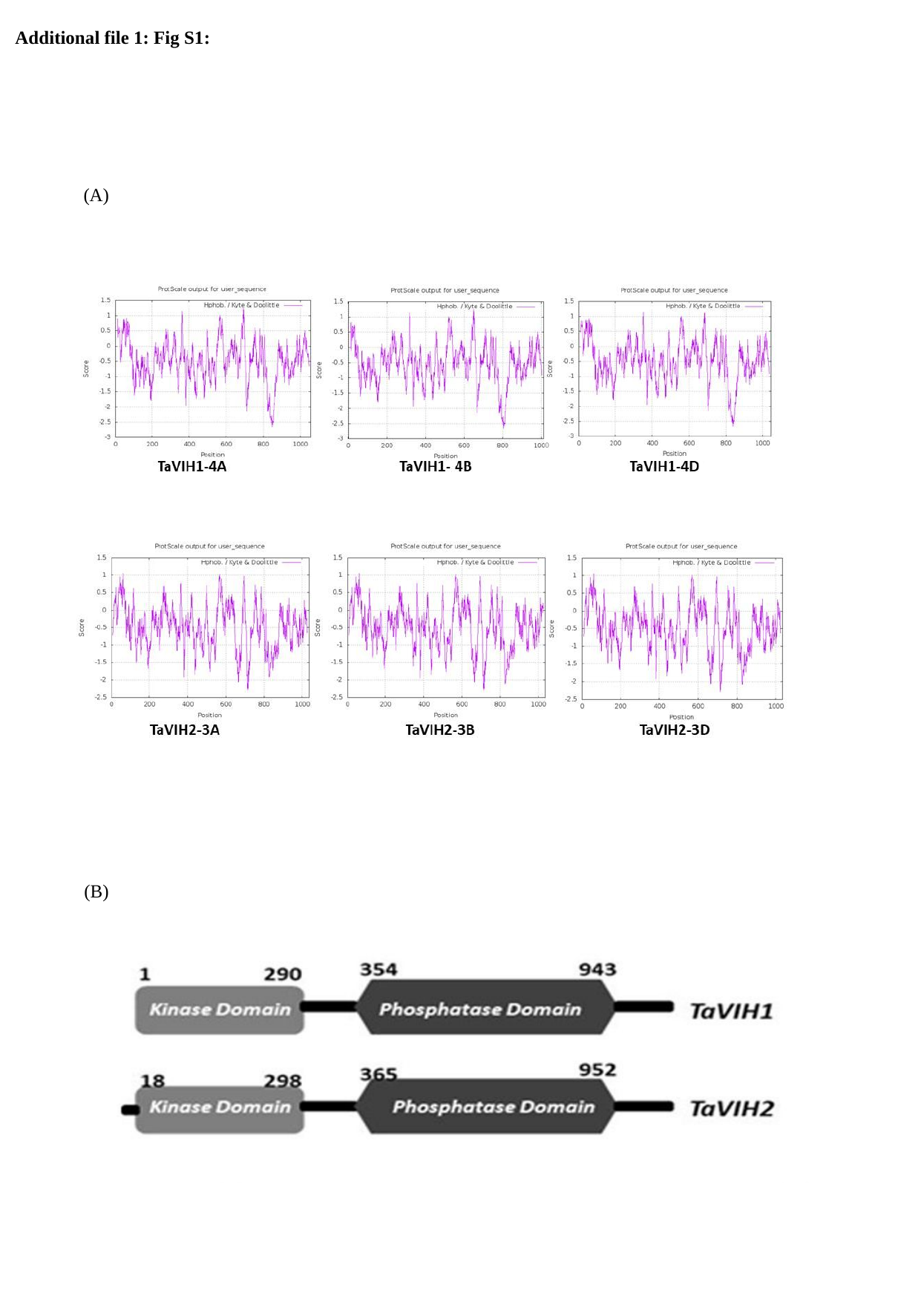

Additional file 1: Fig S1:
(A)
0
(B)

Supplement: Supplementary file 1 — Additional file 1: Fig. S1: Kyte-Doolittle Hydropathy plots and conserved domains of wheat VIH proteins. (A) Kyte-Doolittle hydropathy plots with the positive values indicating the hydrophobic domains and negative values represent hydrophilic regions of the amino acid residues. The hydropathy profile for proteins was calculated according to Kyte and Doolittle, 1982. (B) Schematic representation of domain architecture of TaVIHs deduced from CDD database: light gray rectangles indicate ATP Grasp/RimK Kinase domain and dark gray colored hexagon corresponds to Histidine Phosphatase superfamily. [file 12915_2021_1198_MOESM1_ESM.pptx]

## Slide 1
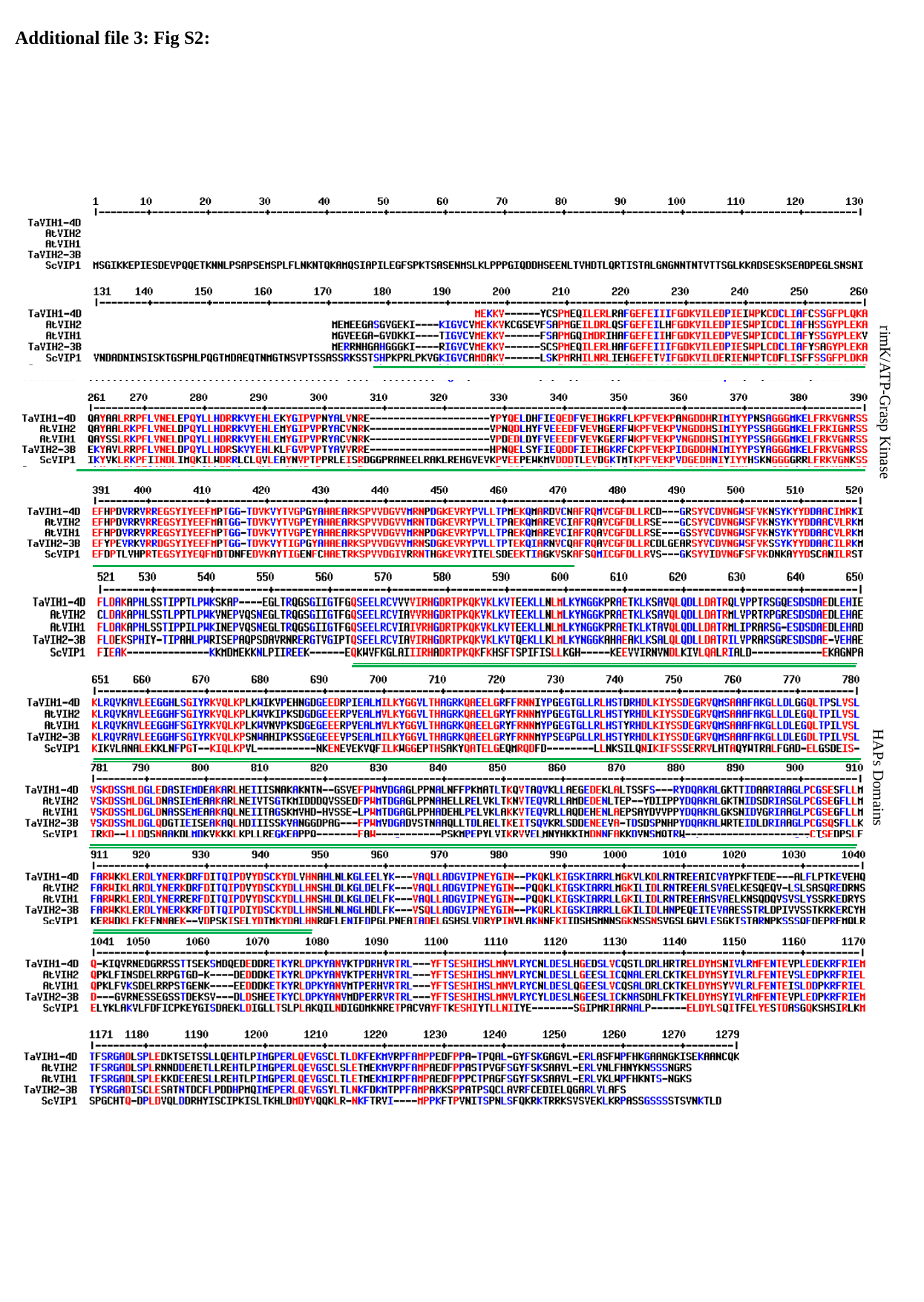

Additional file 3: Fig S2:
rimK/ATP-Grasp Kinase
HAPs Domains

Supplement: Supplementary file 3 — Additional file 3: Fig. S2: Multiple Sequence Alignment (MSA) of different VIH/Vip protein sequences (TaVIH1, TaVIH2, AtVIH1, AtVIH2 and ScVIp1). The red sequence shows high conservation of the amino acids. The single green line indicates rimK/ATP-grasp kinase domain, and the double green line indicates Histidine Phosphatase Domains (HAPs). [file 12915_2021_1198_MOESM3_ESM.pptx]

## Slide 1
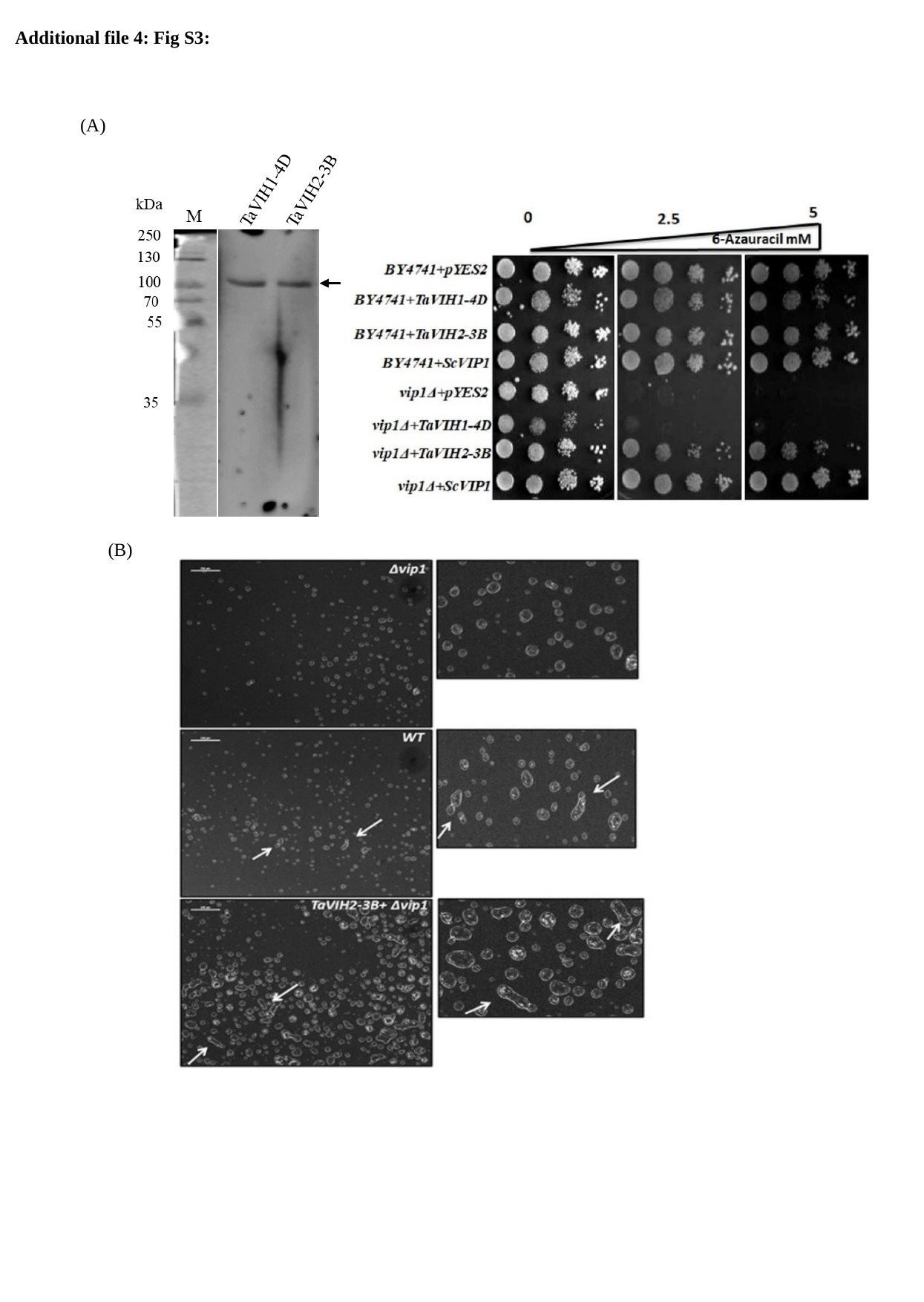

Additional file 4: Fig S3:
(A)
(B)

Supplement: Supplementary file 4 — Additional file 4: Fig. S3: Yeast complementation assays of wheat VIHs. (A) Total protein was extracted from yeast cell transformed with TaVIH1-4D (C-myc tag) and TaVIH2-3B (C-myc tag) and Western analysis was done (left panel). Representative image of spotting assay performed on SD-Ura plates containing 1% raffinose, 2% galactose and supplemented with 0, 2.5 and 5 mM of 6-azauracil (right panel). The wild type BY4741 and vip1Δ strains were transformed with respective constructs using Li-acetate method. Representative images were taken 4 days after the spotting assay was performed. Similar results were obtained with three independent repeats. (B) Filamentous growth assays were observed for wild type yeast (WT), yeast mutant- vip1Δ with empty pYES2 (vip1Δ) and TaVIH2-3B complementation in vip1Δ- (TaVIH2-3B+ Δvip1). Pictures were taken 20 days post-incubation. [file 12915_2021_1198_MOESM4_ESM.pptx]

## Slide 1
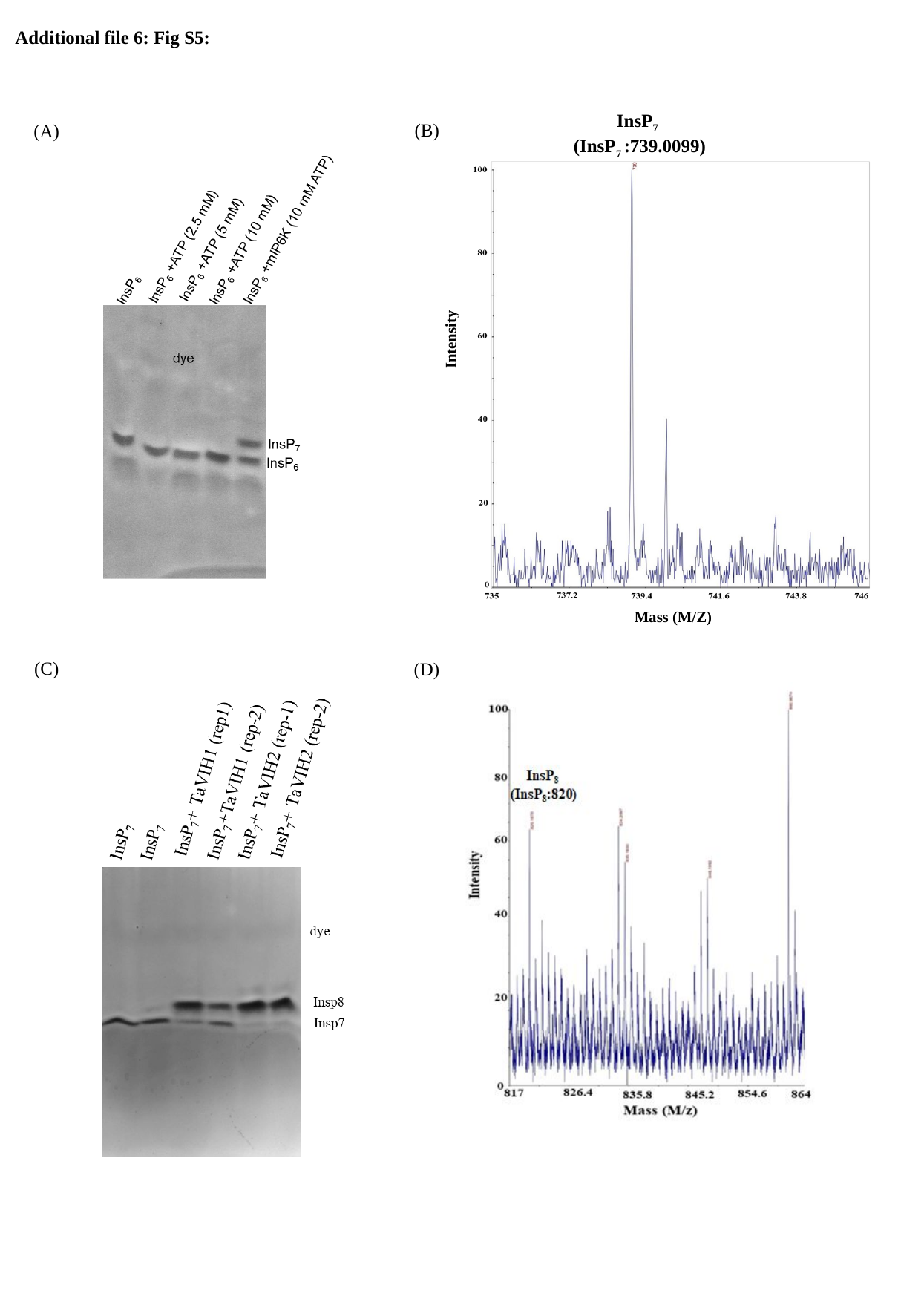

Additional file 6: Fig S5:
InsP7
(InsP7 :739.0099)
Intensity
Mass (M/Z)
(B)
(A)
(C)
(D)

Supplement: Supplementary file 6 — Additional file 6: Fig. S5: (A) PAGE gel (33%) analysis of mIP6K1 generated product by staining with Toluidine Blue. Substrate InsP6 without and with ATP (2.5, 5 and 10 mM) was used as a control. The product InsP7 was generated using mIP6K1 and InsP6 as a substrate (last lane). (B) The InsP7 generated by mIP6K1 was eluted from gel and MS analysis was done which indicated a signal at m/z of 740.3 that correspond to mass of InsP7 and matches with the expected generated species. Indicated by arrow. (C) The kinase reactions were performed using 30 ng of TaVIH1-KD, and TaVIH2-KD purified proteins for 9 hr at 28o C. (D) MALDI-ToF MS analysis of synthesized InsP8 for TaVIH2-3B KD. MS analysis indicated a significant signal at m/z of 820.47 that correspond to the mass of InsP8. Indicated by arrow. [file 12915_2021_1198_MOESM6_ESM.pptx]

## Slide 1
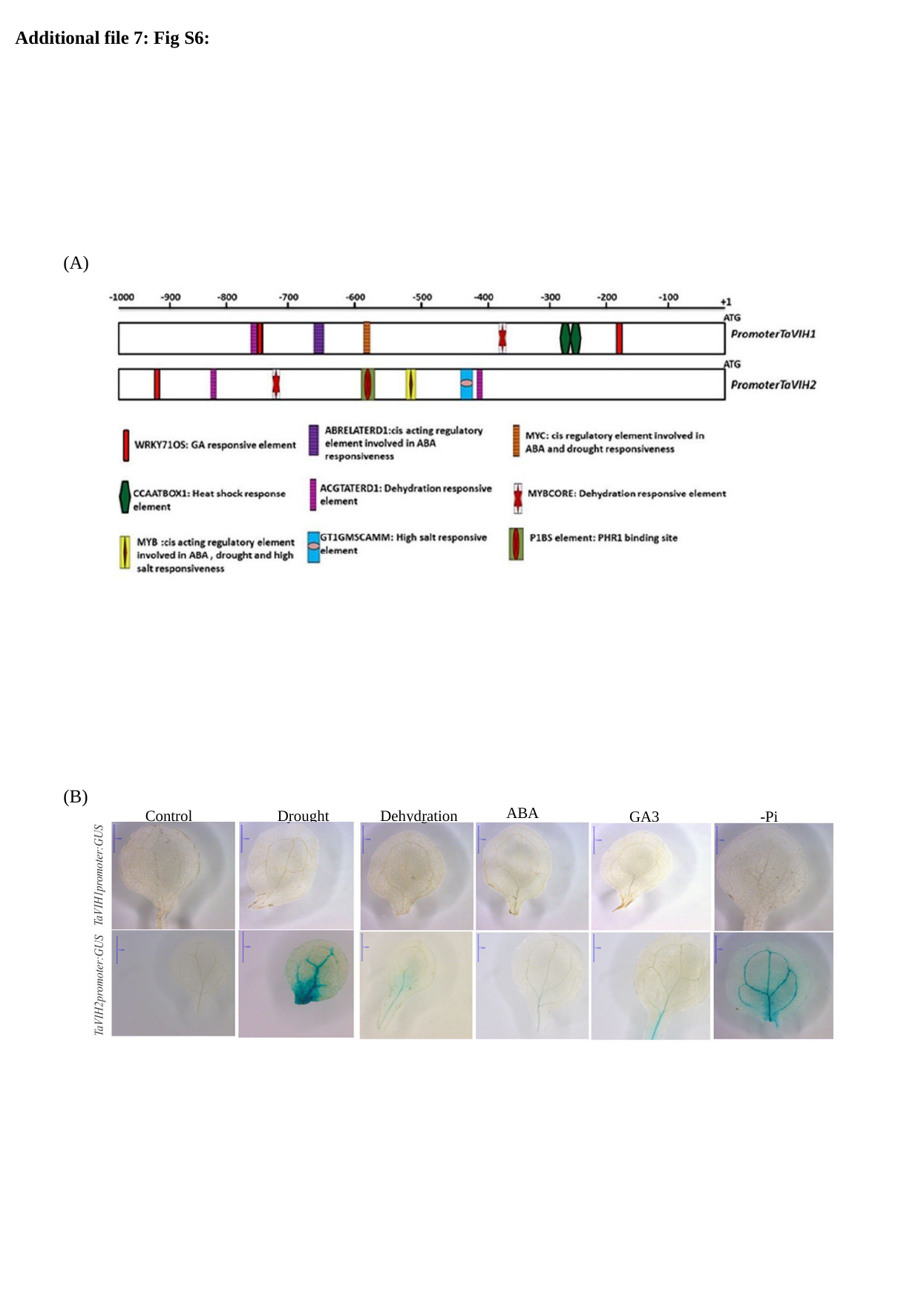

Additional file 7: Fig S6:
(A)
(B)
ABA
Control
Dehydration
Drought
GA3
-Pi

Supplement: Supplementary file 7 — Additional file 7: Fig. S6: Hormonal and abiotic stress response of TaVIH genes promoter. (A) Cis-element analysis of VIH1 and VIH2 promoters (~ 1 kb) Multiple stress related domains are represented in a schematic form. (B) Representative images for histochemical GUS assay performed against different stresses for promTaVIH1:GUS and promTaVIH2:GUS transgenic lines raised in Arabidopsis thaliana Col-0 background. Two-week-old seedlings selected positive against hygromycin selection on 0.5XMS agar plates were subjected to respective treatments: drought (20% PEG), dehydration (1 hr air drying), ABA (100 μM), GA3 (20 μM) and Pi-deficiency (0.5X MS medias without KH2PO4). Seedlings with or without treatment (control) were stained overnight in GUS staining solution and photographed using Leica stereomicroscope at 6.3X magnification. [file 12915_2021_1198_MOESM7_ESM.pptx]

## Slide 1
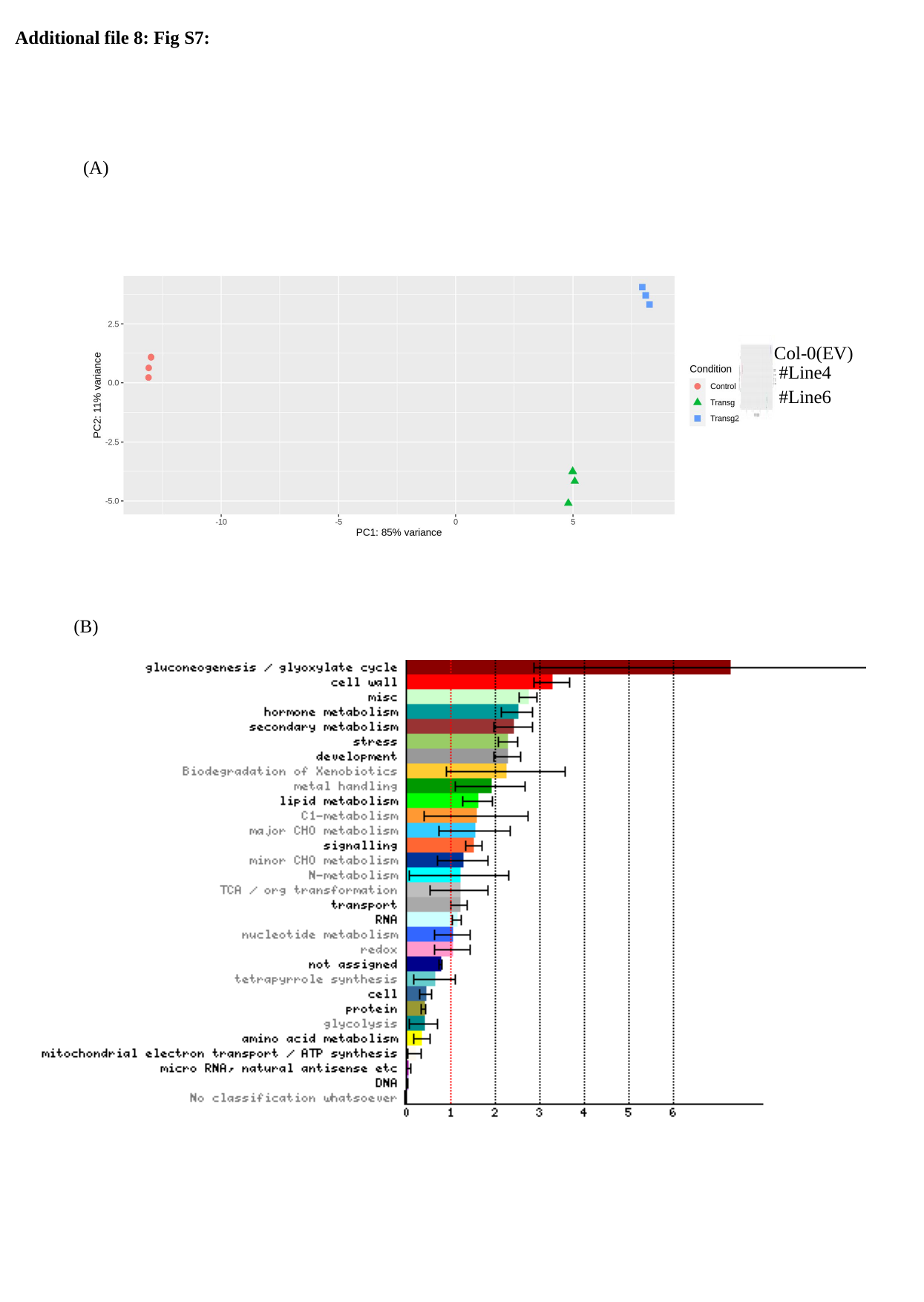

Additional file 8: Fig S7:
(A)
Col-0(EV)
#Line4
#Line6
(B)

Supplement: Supplementary file 8 — Additional file 8: Fig. S7: RNAseq analysis of transgenic Arabidopsis. (A) PCA analysis of the RNAseq for control (Col-0 (Ev)) and two transgenic Arabidopsis lines. (B) Map man analysis of the genes those are consistently represented in the two transgenic Arabidopsis lines with overexpressing TaVIH2-3B. [file 12915_2021_1198_MOESM8_ESM.pptx]

## Slide 1
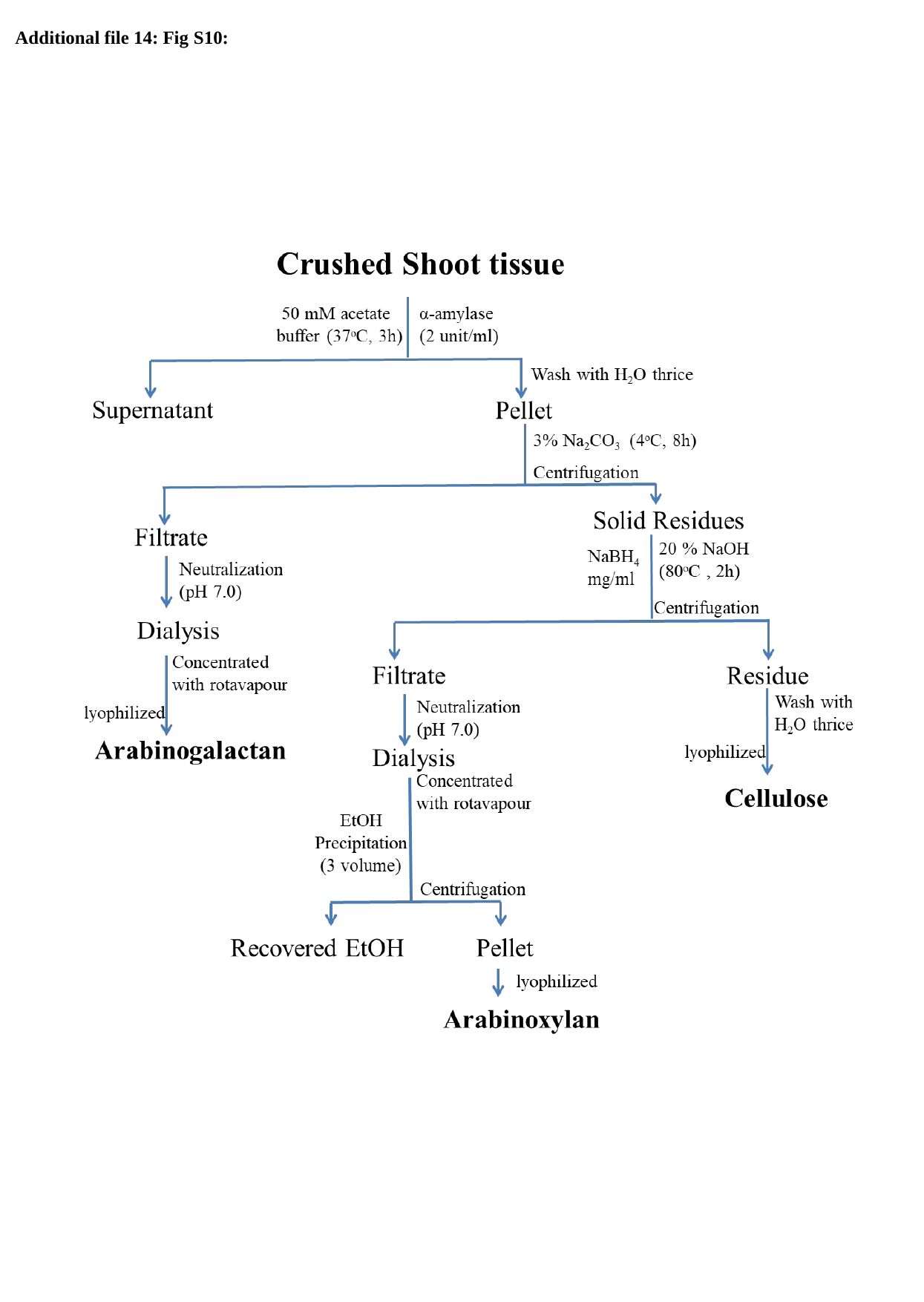

Additional file 14: Fig S10:

Supplement: Supplementary file 14 — Additional file 14: Fig. S10: Flow representation of the preparation and extraction of polysaccharides (Arabinogalactans, Arabinoxylans and Cellulose) form the shoots of Arabidopsis. [file 12915_2021_1198_MOESM14_ESM.pptx]

## Slide 1
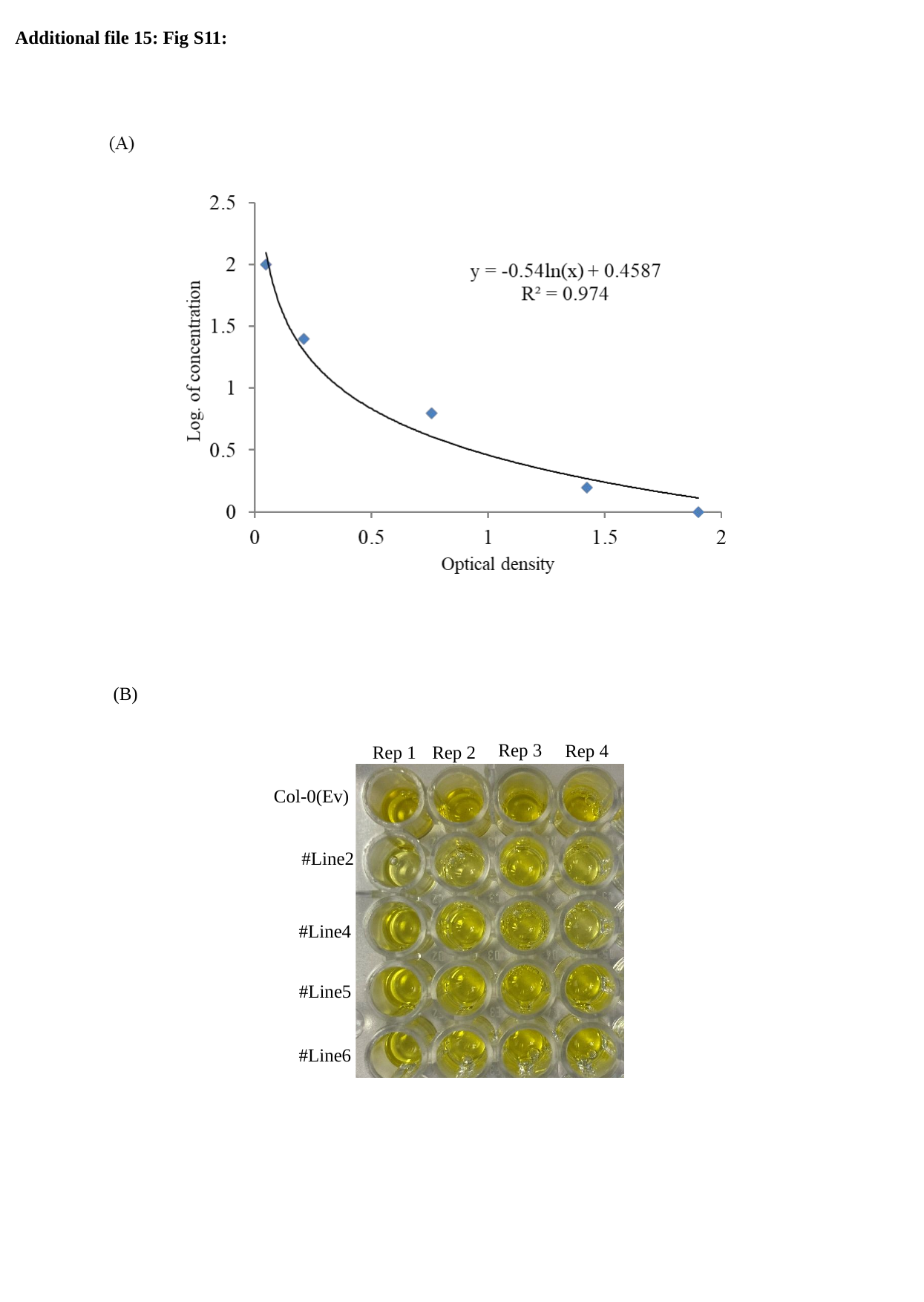

Additional file 15: Fig S11:
(B)
Rep 3
Rep 4
Rep 1
Rep 2
Col-0(Ev)
#Line2
#Line4
#Line5
#Line6

Supplement: Supplementary file 15 — Additional file 15: Fig. S11: Standard graph for ABA measurement in plant leaves samples. (A) Y-axis indicates Log of concentration and X-axis indicates the optical density. Data was linearised by plotting the log of the target antigen concentrations versus the log of the OD and the best fit line was determined by regression analysis. (B) Panel showing the color development for the quantitation of the ABA in different leaf samples, OD was taken at 420 nm. [file 12915_2021_1198_MOESM15_ESM.pptx]
