# Supplementary material for: Wheat inositol pyrophosphate kinase TaVIH2-3B modulates cell-wall composition and drought tolerance in Arabidopsis
Source: BMC Biol. 2021 Dec 11;19:261. doi: 10.1186/s12915-021-01198-8 (PMC8665518; doi:10.1186/s12915-021-01198-8)
Supplement: Supplementary file 5 — Additional file 5: Fig. S4: Protein purification and western analysis of wheat TaVIH1-KD and TaVIH2-KD. The molecular weight is around 40 kDa . Both the VIH proteins (VIH1 and VIH2) were expressed and purified as mentioned in the Methods section, and the expression was confirmed by the Western analysis using His-antibody. [file 12915_2021_1198_MOESM5_ESM.pptx]

## Slide 1
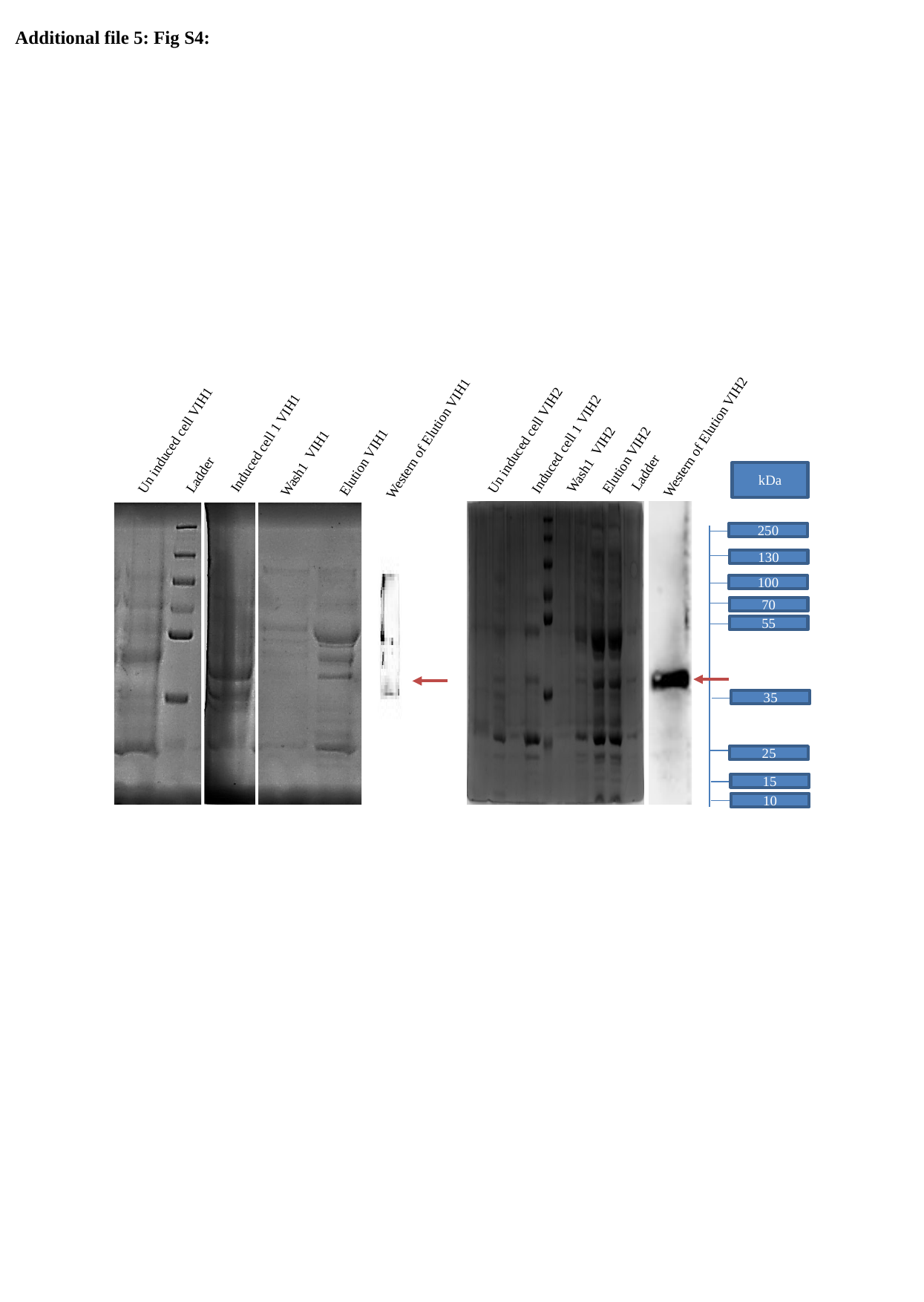

Additional file 5: Fig S4:
Western of Elution VIH2
Un induced cell VIH2
Induced cell 1 VIH2
Wash1 VIH2
Elution VIH2
Ladder
Western of Elution VIH1
Un induced cell VIH1
Induced cell 1 VIH1
Elution VIH1
Ladder
Wash1 VIH1
kDa
250
130
100
70
55
35
25
15
10
