# Supplementary material for: Wheat inositol pyrophosphate kinase TaVIH2-3B modulates cell-wall composition and drought tolerance in Arabidopsis
Source: BMC Biol. 2021 Dec 11;19:261. doi: 10.1186/s12915-021-01198-8 (PMC8665518; doi:10.1186/s12915-021-01198-8)
Supplement: Supplementary file 10 — Additional file 10: Fig. S8: qRT-PCR validation of selected genes from the Col-0(Ev), #Line4 and #Line6. A total of 2 μg of RNA (DNA free) was used for cDNA synthesis and qRT-PCR was performed using gene specific primers (Supplementary Table S4). Ct values were normalized against wheat ARF1 as an internal control. [file 12915_2021_1198_MOESM10_ESM.pptx]

## Slide 1
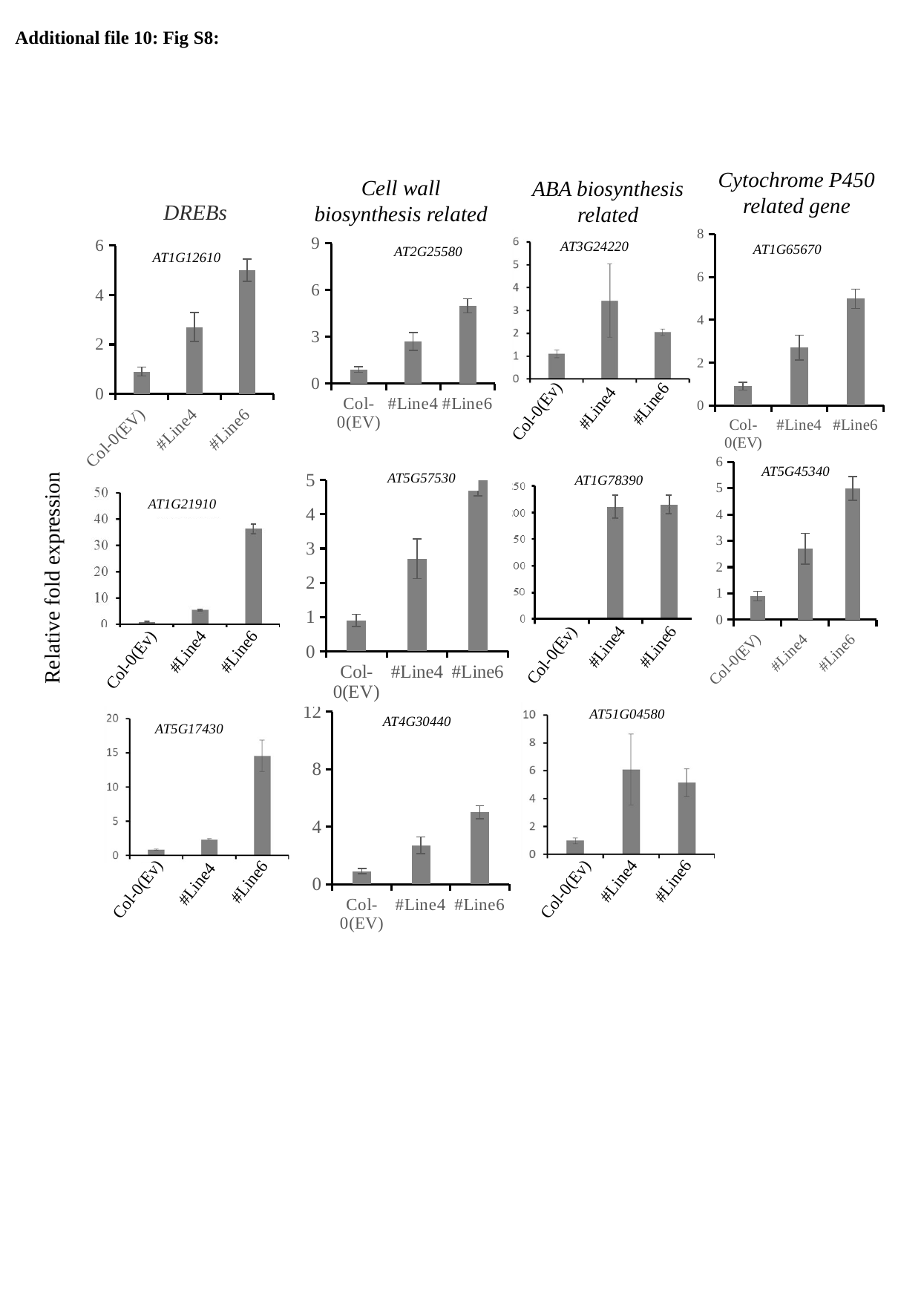

Additional file 10: Fig S8:
Cytochrome P450 related gene
Cell wall biosynthesis related
ABA biosynthesis related
DREBs
### Chart
| Category | |
|---|---|
| Col-0(EV) | 0.9 |
| #Line4 | 2.7 |
| #Line6 | 4.99 |
### Chart
| Category | |
|---|---|
| Col-0(EV) | 0.9 |
| #Line4 | 2.7 |
| #Line6 | 4.99 |
### Chart
| Category | |
|---|---|
| Col-0(EV) | 0.9 |
| #Line4 | 2.7 |
| #Line6 | 4.99 |
Col-0(Ev)
#Line6
#Line4
AT3G24220
AT1G65670
AT2G25580
AT1G12610
### Chart
| Category | |
|---|---|
| Col-0(EV) | 0.9 |
| #Line4 | 2.7 |
| #Line6 | 4.99 |AT5G45340
AT5G57530
### Chart
| Category | |
|---|---|
| Col-0(EV) | 0.9 |
| #Line4 | 2.7 |
| #Line6 | 4.99 |AT1G78390
AT1G21910
Relative fold expression
Col-0(Ev)
#Line4
#Line6
Col-0(Ev)
#Line4
#Line6
### Chart
| Category | |
|---|---|
| Col-0(EV) | 0.9 |
| #Line4 | 2.7 |
| #Line6 | 4.99 |AT51G04580
Col-0(Ev)
#Line4
#Line6
Col-0(Ev)
#Line6
#Line4
AT5G17430
AT4G30440
