# Supplementary material for: Wheat inositol pyrophosphate kinase TaVIH2-3B modulates cell-wall composition and drought tolerance in Arabidopsis
Source: BMC Biol. 2021 Dec 11;19:261. doi: 10.1186/s12915-021-01198-8 (PMC8665518; doi:10.1186/s12915-021-01198-8)
Supplement: Supplementary file 12 — Additional file 12: Fig. S9: Expression patterns of TaVIH gene homoeologous in different tissues and stress conditions. RNAseq datasets of (A) Tissues and developmental stages (B) Abiotic (phosphate starvation, heat and drought stress) and (C) Biotic stress conditions were used. The expression values were obtained from expVIP database in the form of TPM values and ratios of stressed to control condition were used to generate heatmaps using MeV software. Green and red colors represent down-regulation and up-regulation of the genes in the specific stresses, as shown by the color bar. [file 12915_2021_1198_MOESM12_ESM.pptx]

## Slide 1
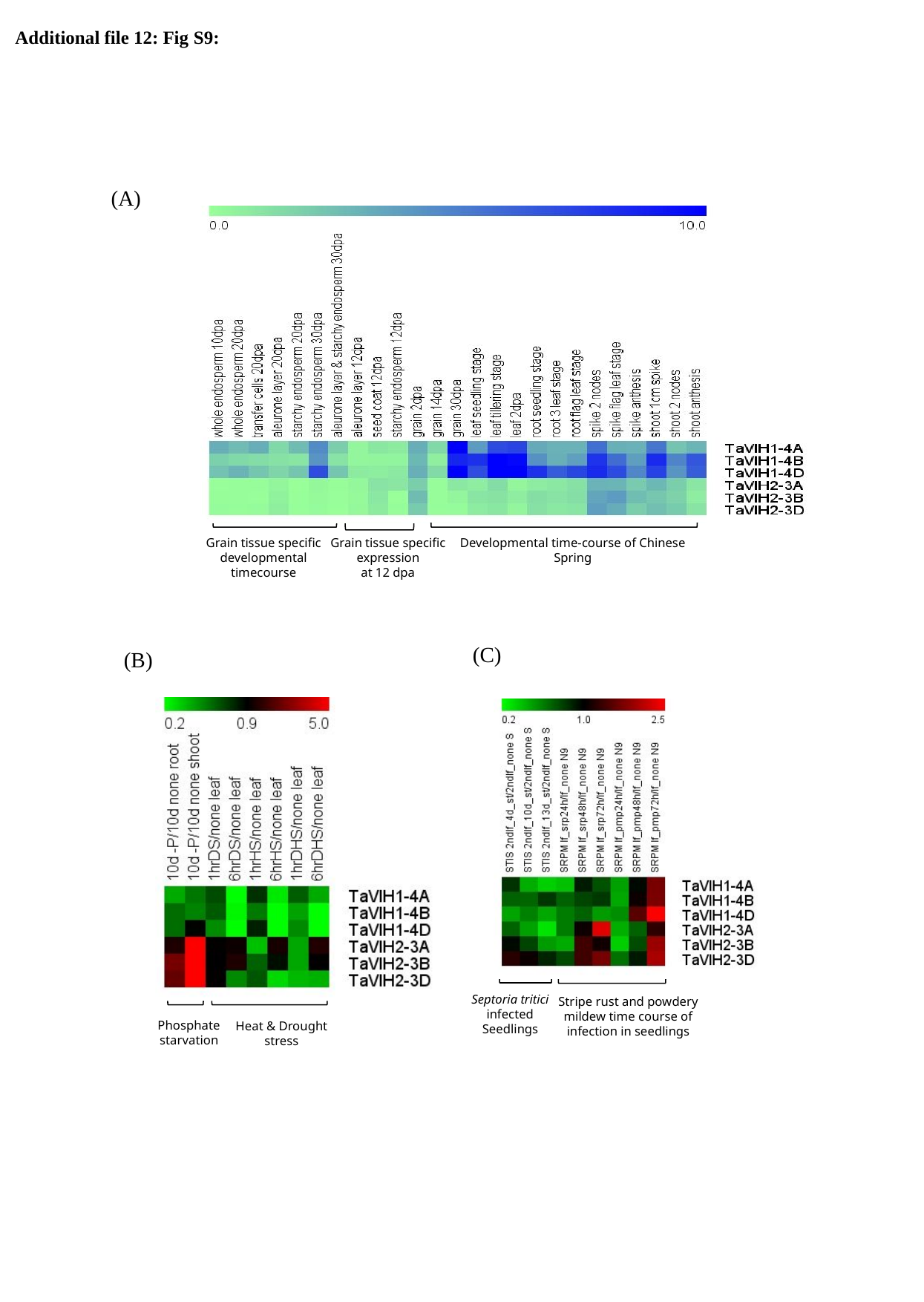

Additional file 12: Fig S9:
(A)
Grain tissue specific developmental timecourse
Grain tissue specific expression
at 12 dpa
Developmental time-course of Chinese Spring
(C)
(B)
Phosphate starvation
Heat & Drought stress
Septoria tritici infected Seedlings
Stripe rust and powdery mildew time course of infection in seedlings
